# Towards in vivo imaging of functionally active 5-HT_1A_ receptors in schizophrenia: concepts and challenges

**DOI:** 10.1038/s41398-020-01119-3

**Published:** 2021-01-07

**Authors:** Oriane Razakarivony, Adrian Newman-Tancredi, Luc Zimmer

**Affiliations:** 1 Université de Lyon, Lyon Neuroscience Research Center, INSERM, CNRS, Lyon, France; 2 Hospices Civils de Lyon, Lyon, France; 3 Neurolixis, Castres, France; 4 CERMEP-Imagerie du Vivant, Bron, France; 5 French National Institute for Nuclear Science and Technology, CEA Saclay, Gif-sur-Yvette, France

**Keywords:** Schizophrenia, Biomarkers

## Abstract

The serotonin 5-HT_1A_ receptor has attracted wide attention as a target for treatment of psychiatric disorders. Although this receptor is important in the pharmacological mechanisms of action of new-generation antipsychotics, its characterization remains incomplete. Studies based on in vitro molecular imaging on brain tissue by autoradiography, and more recently in vivo PET imaging, have not yielded clear results, in particular due to the limitations of current 5-HT_1A_ radiotracers, which lack specificity and/or bind to all 5-HT_1A_ receptors, regardless of their functional status. The new concept of PET neuroimaging of functionally active G-protein-coupled receptors makes it possible to revisit PET brain exploration by enabling new research paradigms. For the 5-HT_1A_ receptor it is now possible to use [^18^F]-F13640, a 5-HT_1A_ receptor radioligand with high efficacy agonist properties, to specifically visualize and quantify functionally active receptors, and to relate this information to subjects’ pathophysiological or pharmacological state. We therefore propose imaging protocols to follow changes in the pattern of functional 5-HT_1A_ receptors in relation to mood deficits or cognitive processes. This could allow improved discrimination of different schizophrenia phenotypes and greater understanding of the basis of therapeutic responses to antipsychotic drugs. Finally, as well as targeting functionally active receptors to gain insights into the role of 5-HT_1A_ receptors, the concept can also be extended to the study of other receptors involved in the pathophysiology or therapy of psychiatric disorders.

## Introduction

Serotonin (5-hydroxytryptamine, 5-HT) receptors constitute a large family of seven receptor classes (5-HT_1_ to 5-HT_7_) with 14 different subtypes. Five subgroups are distinguished within the 5-HT_1_ receptor group, i.e., 5-HT_1A_, 5-HT_1B_, 5-HT_1D_, 5-HT_1E_ and 5-HT_1F_ receptors. The 5-HT_1A_ subtype was the first of the serotonin receptors to be sequenced and is nowadays one of the most thoroughly studied. Its distribution within the brain is classically divided into two functional groups: presynaptic autoreceptors which are present at high densities in the midbrain raphe nuclei, and postsynaptic heteroreceptors in other brain regions, notably the hippocampus, the cingulate cortex, the lateral septum and other brain regions that are important for control of mood and cognition. Presynaptic 5-HT_1A_ autoreceptors on serotonergic neurons mediate an important negative feedback influence on neuronal firing, thus eliciting inhibition of 5-HT release. Postsynaptic 5-HT_1A_ receptors are located on different cell types, including GABAergic interneurons and excitatory pyramidal and granule cells. Due to its modulating influence on the whole serotonergic system and its large distribution throughout the brain, the 5-HT_1A_ receptor is considered as one of the most important 5-HT receptor subtypes, and is of primary interest when investigating serotonergic neurotransmission in brain^1,2^.

The 5-HT_1A_ receptor is strongly associated with alterations in mood and emotion, as demonstrated by numerous preclinical and clinical studies^1^. Many of these are focused on depression and anxiety, but compelling evidence also points to an important role of 5-HT_1A_ receptor dysfunction in schizophrenia^3^. This chronic psychiatric disease affects around 20 million people worldwide^4^, creating a substantial global burden of care, with significant morbidity and functional impact^4,5^. Notably, life expectancy in patients with schizophrenia has been estimated to be 10–20 years shorter than in the general population^6^. Although schizophrenia is associated with an imbalance in dopamine transmission, with an hyperactivity in the mesolimbic system and an hypoactivity in the prefrontal cortex, other neurotransmitters, including glutamate, GABA and serotonin, are also involved in its physiopathological process^7,8^. The main symptoms of schizophrenia comprise delusions, hallucinations, disorganization and negative symptoms, i.e., blunted affect or aboulia. Diagnosis is based on the presence of at least two of these main symptoms for 6 months or more, leading to significant functional impairment. Cognitive impairment is a core feature of schizophrenia and may be present at any stage of the disease^9^. Both negative and cognitive symptoms are associated with poor functional outcomes^10^ and are currently not well-managed by available pharmacological treatments. A better understanding of the physiopathology of schizophrenia is thus required to identify and validate pharmacological targets and, ideally, lead to novel therapeutic strategies for patients.

## The involvement of serotonin 5-HT_1A_ receptors in the pharmacology of antipsychotics

Early classical (i.e., typical) antipsychotics (e.g., phenothiazine, butyrophenone, and benzamide derivatives) possess dopamine D_2_ receptor blocking activity and show efficacy for control of positive symptoms. These agents, however, are not very effective for treatment of negative or cognitive symptoms and frequently induce extra-pyramidal side effects. Subsequently, a variety of atypical antipsychotics became available as first-line treatments. On a pharmacological level, these atypical antipsychotics commonly act as antagonists on both the dopaminergic D_2_ receptor and the serotoninergic 5-HT_2A_ receptor, as well as interacting with multiple other receptors^11–13^. For example, antipsychotics such as risperidone or olanzapine display the desired two-fold action (D_2_ and 5-HT_2A_) and exhibit efficacy against positive symptoms with improved activity on negative and cognitive symptoms and reduced side effects, such as extra-pyramidal symptoms (EPS)^11–13^. Nonetheless, there are still unmet needs in the treatment of schizophrenia, including patients who are resistant to treatment with current antipsychotics^14,15^, the need to more efficaciously improve cognitive deficits, alleviate affective disorders (e.g., anxiety and depression) and reduce antipsychotic‐induced EPS, metabolic and endocrine side effects^16^.

It is notable that some atypical antipsychotic drugs that primarily target D_2_ and 5-HT_2A_ receptors, such as risperidone and olanzapine, also indirectly activate 5-HT_1A_ receptors^17^. Other antipsychotics, including clozapine, ziprasidone and quetiapine, act directly as partial agonists at 5-HT_1A_ receptors^18^. Moreover, ‘third generation’ antipsychotics, such as aripiprazole, cariprazine and brexpiprazole, exhibit prominent direct-acting agonist properties at 5-HT_1A_ receptors^19–21^. Indeed, 5‐HT_1A_ receptors are now an important therapeutic target for selection of novel antipsychotics based on a range of observations^12,22^. For example, 5-HT_1A_ receptor activation has been proposed as a target for the development of cognitive enhancers for treatment of cognitive dysfunction in schizophrenia, notably via stimulation of dopamine release in key regions such as the frontal cortex^11,23^. Thus, some authors proposed that verbal memory and executive functioning were improved in schizophrenia patients that received the 5-HT_1A_ partial agonist, tandospirone, in addition to neuroleptic treatment^24,25^. Based on molecular, neurochemical, behavioural and clinical data, it has been suggested that agonism of the 5-HT_1A_ receptor may ameliorate the therapy of negative symptoms and comorbid mood deficits, attenuate cognitive deficits and oppose EPS elicited by D_2_ receptor blockade. Future antipsychotics may therefore be identified that possess an optimized dual D_2_/5-HT_1A_ mechanism which retains efficacy against positive symptoms via D_2_ antagonism, whilst improving cognitive impairment and negative symptoms associated with schizophrenia and functional outcome by activation of 5-HT_1A_ receptors^26,27^.

In addition to pharmacological factors such as those described above, genetic factors could also influence the role of 5-HT_1A_ receptors in schizophrenia^28^. Indeed, accumulating evidence indicates that a common 5-HT_1A_ gene promoter single-nucleotide polymorphism (SNP rs6295, C-1019G), which occurs in about 5–10% in Oriental populations and 40% in Caucasian population^29^, leads to changes in 5-HT_1A_ receptor function that markedly affect vulnerability to psychiatric disorders, including schizophrenia^30^. Thus, several studies highlighted the correlation between 5-HT_1A_ receptor polymorphisms and the presence of schizophrenia symptoms and their response to treatments^31–33^. This single-nucleotide polymorphism is proposed to have a relevant impact on 5-HT_1A_ receptor density and/or activity. In raphe nuclei, the C-G change linked to the polymorphism impairs the binding of nuclear proteins (e.g., Deaf1) to a palindromic DNA sequence located at this polymorphism. As a result, the rs6295 G allele is associated with a reduced efficiency of Deaf-1 binding, expressed in an increase of 5-HT_1A_ autoreceptor density in the raphe nucleus. In contrast, the 5-HT_1A_ receptor expression is proposed to be decreased on the postsynaptic part, such as in the prefrontal cortex. This lowered postsynaptic 5-HT_1A_ receptor density is linked to a reduced serotonergic signal transduction, which may in turn reduce dopamine release in the prefrontal cortex^34^. As a consequence, this serotonin imbalance may have implications regarding response to antipsychotics (notably as concerns negative symptoms) and treatment options for patients with schizophrenia^29^. Taken together, these findings reinforce the assertion that the 5-HT_1A_ receptor is a relevant target for treatment of schizophrenia and other psychiatric disorders.

## The exploration of 5-HT_1A_ receptors in schizophrenia by molecular imaging and its contradictory results

The development of molecular imaging techniques with selective radioligands allows direct measurement of serotonin receptors in vitro on brain tissue by autoradiography, or in vivo, in the living human brain by positron emission tomography (PET). Several compounds have been radiolabelled for studies of the 5-HT_1A_ receptor. The most commonly used radioligand for in vitro studies is [^3^H]-8-OH-DPAT and the most frequent PET radiopharmaceuticals for quantification of 5-HT_1A_ receptors in the living human brain are [^11^C]-WAY100635 and [^18^F]-MPPF.

### Post-mortem studies and their shortcomings

The first exploration of the 5-HT_1A_ receptor densities in the brain of schizophrenic patients was carried out by in vitro autoradiography on post-mortem brain tissues. The aim was to investigate the role of the 5-HT_1A_ receptor in the pathophysiology of schizophrenia. Other studies followed and, in total, there have been eleven post-mortem studies exploring the G-protein-coupled serotonin 5-HT_1A_ receptor in patients with schizophrenia compared to healthy controls by homogenate or in situ autoradiographic binding techniques (for review, see ref. ^35^). Although the majority of these post-mortem studies described an increase of 5-HT_1A_ receptor densities in various cortical regions, several articles reported contradictory results. Six studies showed an increase in 5-HT_1A_ receptor density in the prefrontal cortex (PFC) of patients with schizophrenia compared with healthy controls^36–41^ whereas three did not^42–44^; one study found a significant increase in the temporal cortex^38^ and two did not^36,44^. Other contradictory results mainly concerned the anterior cingulate cortex and the hippocampus^36–38,40,44,45^. One study showed an increase in 5-HT_1A_ receptor density in the cerebellum of patients with schizophrenia compared with controls^46^. No studies found any decrease of the 5-HT_1A_ receptors in any brain region in patients with schizophrenia compared to controls. Overall, meta-analysis from Selvaraj and colleagues^35^ found that the pooled results were in favour of a significant increase in 5-HT_1A_ receptors in the prefrontal cortex of patients with schizophrenia compared to healthy controls, with an effect size of 0.60 (CI: 0.17–1.03, *p* = 0.007)^35^. For detailed results, see Table 1.

**Table 1:** Post-mortem studies exploring 5-HT_1A_ receptors in schizophrenia.

Reference	Schizophrenia group	Control group	Radiotracer	Region of interest	Main result
^36^	*n* = 13; mean age 55 +/−16 yrs; 8 M/5 F; suicide 2/13; antipsychotics 12/13	*n* = 15; mean age 59 + /−15 yrs; 9 M/6 F; suicide 2/15	[^3^H]-8-OH-DPAT	DLPFC (BA 46)	Increase (23%)
				ACC (BA 24)	NS
				STG; striatum, cortex; hippocampus	NS
^37^	*n* = 9; mean age 53 +/−15 yrs; 4 M/5 F; suicide 1/9; antipsychotics 8/9	*n* = 9; mean age 58 +/−17 yrs; 5 M/4 F; no suicide	[^3^H]-WAY-100635	DLPFC (BA 46)	Increase (17%)
				ACC (BA 24)	NS
^43^	*n* = 10; mean age 60 +/−3.7 yrs; 7 M/3 F; no suicide; antipsychotics 8/10	*n* = 10; mean age 60 +/−3.8 yrs; 7 M/3 F; no suicide	[^3^H]-8-OH-DPAT	DLPFC (BA 8, 9, 10)	NS
^42^	*n* = 8; 5 M/3 F; no suicide; antipsychotics 8/8	Healthy controls: *n* = 8; 5 M/3 F; no suicide Bipolar I disorder: *n* = 8; 4 M/4 F; *n*o suicide; antipsychotics 5/8.	[^3^H]-8-OH-DPAT	Frontal cortex (BA 9,10, 40, 46)	NS
^40^	*n* = 10; mean age 75.4 +/−1.5 yrs; 6 M/4 F; antipsychotics 4/10; no suicide	*n* = 13; mean age 63.3 +/−3.3 yrs; 7 M/4 F; no suicide	[^3^H]-8-OH-DPAT	ACC (BA 24)	Increase
				PFC (BA 6,9a and 44)	Increase
				Motor cortex; sensori-motor cortex; infra-parietal cortex; PCC	NS
^38^	*n* = 10; mean age 67.3 +/−3.8 yrs; 6 M/4 F; antipsychotics 5/10	*n* = 11; mean age 63.6 +/−4 yrs; 4 M/7 F	[^3^H]-8-OH-DPAT	PFC (BA 10)	Increase (40%)
				Temporal cortex (BA 22)	Increase (60%)
				Parietal cortex; motor cortex; occipital cortex; cingulum; amygdala; hippocampus	NS
^44^	*n* = 10; mean age 49 +/−18 yrs; 7 M/3 F; suicide 4/10; antipsychotics 7/10	Control group: *n* = 8; mean age 68 +/−15 yrs; 4 M/4 F; no suicide Suicide/affective illness group: *n* = 8; mean age 38 +/−13 yrs; 5 M/3 F; medication 4/10	[^3^H]-8-OH-DPAT	Motor cortex (BA 4)	Increase
				ACC (BA 24)	Increase
				PCC (BA 23)	Increase
				Hippocampus	Increase
				Temporal cortex; striatum; frontal (BA9)	NS
^45^	*n* = 20; mean age 47.35 +/−3.7 yrs; 11 M/9 F; suicide 5/20; antipsychotics 20/20	*n* = 20; mean age 46.2 +/−3.4 yrs; 13 M/7 F; no suicide	[^3^H]-8-OH-DPAT	Hippocampus	NS
^39^	*n* = 12; mean age 59 +/−6 yrs; 9 M/3 F; suicide 4/10; antipsychotics 10/12	*n* = 18; mean age 63 +/−2 yrs; 13 M/5 F; no suicide	[^3^H]-8-OH-DPAT	Medial orbital gyrus	Increase
				Straight gyrus	Increase
				Olfactory sulcus	Increase
^46^	*n* = 19; mean age 54.3/−1.4 yrs; 14 M/5 F; antipsychotics 19/19	*n* = 16; mean age 71.5 +/−3.9 yrs; 10 M/6 F	[^3^H]-8-OH-DPAT	Cerebellar vermis lobules	Increase
^41^	*n* = 12; mean age 37.8 + /−2.9 yrs; 7 M/5 F; suicide 5/12; antipsychotics 10/12	*n* = 12; mean age 37.1 +/−3.4 yrs; 10 M/1 F; no suicide; medication 3/10	[^3^H]-8-OH-DPAT	PFC (BA10)	Increase (79%)

*ACC* anterior cingulate cortex, *BA* Brodman’s area, *DLPFC* dorsolateral prefrontal cortex, *F* females, *M* men, *NS* non-significant, *PFC* prefrontal cortex, *PCC* posterior cingulate cortex, *STG* superior temporal gyrus, *yrs* years.

### PET studies and their shortcomings

There have been few in vivo 5-HT_1A_ positron emission tomography (PET) imaging studies in schizophrenia patients. PET imaging theoretically has great advantages over the in vitro approach. Indeed, in vivo imaging allows the exploration of live patients, allowing for possible longitudinal follow-up. Moreover, PET imaging permits exploration of the entire brain, potentially revealing regions of interest not examined in the brain tissue sections available in brain banks. However, results from PET studies on the 5-HT_1A_ receptor in schizophrenia were mainly non-significant^47,48^ or contradictory^49,50^. For example, a PET study focused on 5-HT_1A_ receptor availability in schizophrenia patients treated with ziprasidone and found no significant difference in their distribution before or after receiving medication but found a significant association between 5-HT_1A_ binding and improvement in negative symptoms^51^. For detailed results, see Table 2.

**Table 2:** PET imaging studies exploring 5-HT_1A_ receptors in schizophrenia.

Reference	Schizophrenia group	Control group	Radiotracer	Reference region	Main result
^47^	Non-clozapine group: *n* = 11; 11M/0F; mean age 41.5 +/−12 yrs Clozapine group: *n* = 11; 11M/0F; mean age 36.9 +/−10.5 yrs	*n* = 11; mean age 37.3 +/−8 yrs; 11M/0F	[^11^C]-WAY100635	Cerebellar hemisphere regions	NS
^48^	*n* = 22; mean age 32 +/−10 yrs; 16M/6F; 14 drug free/8 drug naive; 15 schizophrenia/7 schizoaffective disorder diagnosis	*n* = 18; mean age 32 + /−8 yrs; 15M/3F	[^11^C]-WAY100635	None (Kinetic modelling with arterial input modelling strategy)	NS
^49^	Drug naive first psychosis episode patients: *n* = 14; mean age 26 +/−5 yrs; 8M/6F	*n* = 14; mean age 28 +/−5 yrs; 6M/8F	[^11^C]-WAY100635	Cerebellum	Increase (+7.1% +/-6.4%; *p* = 0.02)
^50^	*n* = 11; 9 M/2 F; mean age 31.1 +/−8.7 yrs; 9 M/2 F; 3 drug free/8 drug naive; 5 schizophrenia/6 schizophreniform diagnosis	*n* = 22; mean age 31 +/−8.5 yrs; 18M/4F	[^11^C]-WAY100635	Cerebellum	Decrease in the amygdala (−19%; *p* = 0.023)

*F* female, *M* male, *NS* non-significant, *yrs* years.

It is striking that although molecular imaging studies of the 5-HT_1A_ receptor in schizophrenia report disparate results, none of them have yet confirmed post-mortem findings. The contrast between in vitro and in vivo findings may be attributable to several factors, such as variations in the sample populations (age, duration of illness, pharmacological treatments…) but also to the methodological approaches. Thus, there are significant pharmacological differences between the radiotracers used for quantification of in vitro and in vivo studies. The 5-HT_1A_ receptor can exist in a high (G-protein-coupled, functionally active) or low (G-protein-decoupled, non-functionally active) affinity state with respect to endogenous binding. As mentioned before, most post-mortem studies have used the agonist [^3^H]-8-OH-DPAT, which binds with high affinity only to receptors in high-affinity state and it also has limited specificity, binding to both 5-HT_1A_ and 5-HT_7_ receptors^52^. On the contrary, PET studies used antagonist radiotracers, [^11^C]-WAY100635 or [^18^F]-MPPF, that are more specific (although WAY100635 also has nanomolar range binding affinity for dopamine D4 receptors, rarely taken into account^53^), and that bind with comparable affinity to 5-HT_1A_ receptors in both high- and low-affinity states^54^. While these characteristics are well known to neuropharmacologists, they are seldom given consideration in the nuclear medicine and neuroscience communities.

## The concept of functionally active 5-HT_1A_ receptor imaging

### Principle: antagonist versus agonist

The vast majority of PET radiopharmaceuticals currently used for receptor neuroimaging are antagonists. However, about 30 agonists have been used for PET imaging of various G-protein-coupled receptors: adrenergic, dopaminergic, serotonergic, muscarinic, cannabinoid, and opioid receptors^55–57^. Among them, a dozen of PET agonist radiotracers were developed and translated to humans for these receptors, but very few studies^58–60^ directly compared the in vivo density of receptors targeted by an agonist versus an antagonist radiotracer in the same subject (in these cases, animal models). Several interpretations have been given to in vivo binding differences based on the pharmacological agonist/antagonist properties of the ligands. The internalization of certain receptors has been evoked, as well as the modification of their allosteric conformations, or the influence of the extracellular concentration of the endogenous neurotransmitter, all of which could influence differently the binding of an antagonist and an agonist for the same family of receptors (see ref. ^56^ for a review on this subject).

**Fig. 1:**
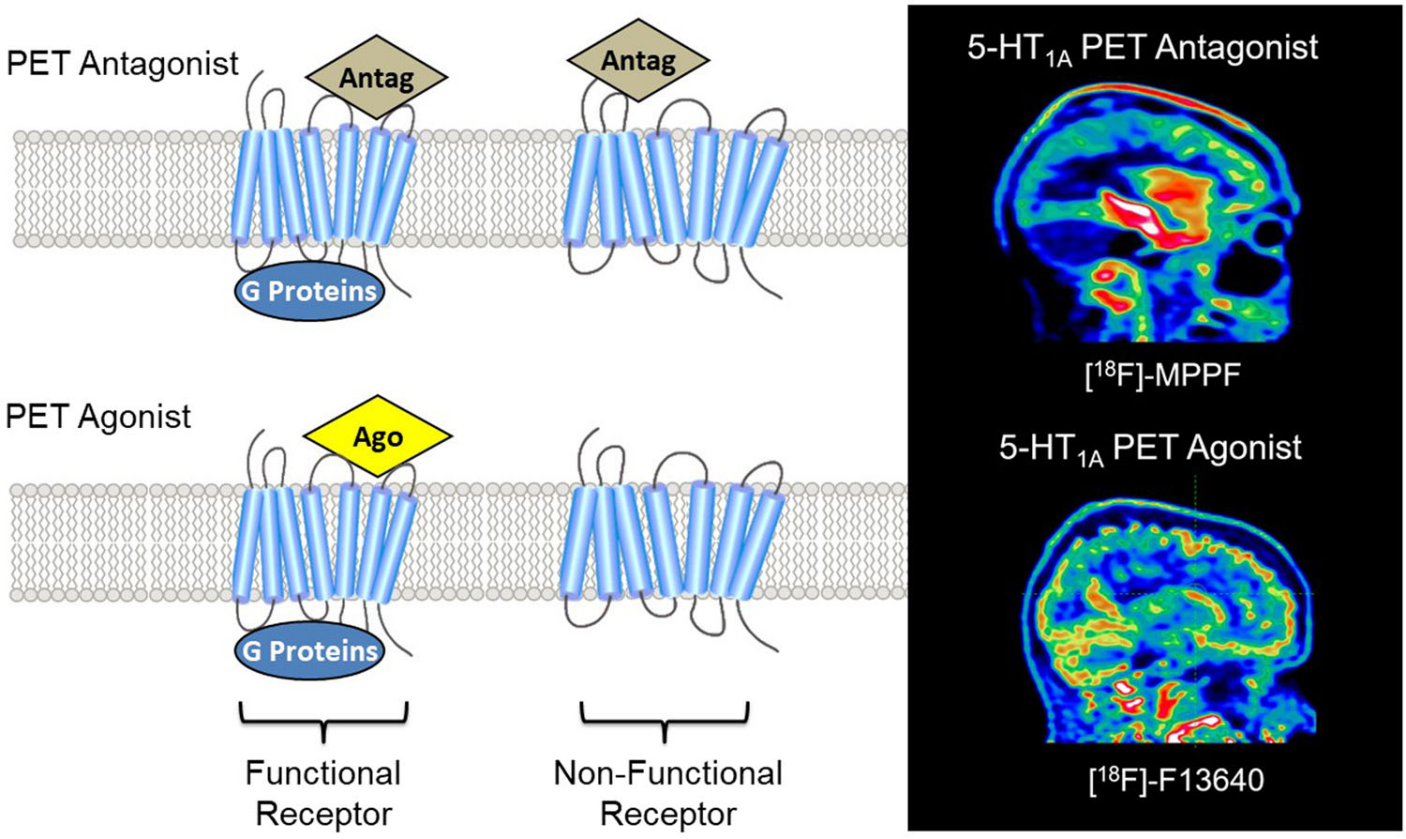
Comparison of PET antagonist and agonist radiotracer binding. The antagonist radiotracer binds to all states of the receptors, i.e. functional (bound to G protein) and non-functional (uncoupled form) whereas the agonist only binds to functional receptors (bound to G protein). As a result, the binding pattern at 5-HT_1A_ receptors differs significantly between the antagonist [^18^F]-MPPF (binding in hippocampus, cingulate cortex and raphe nuclei) and the agonist [^18^F]-F13640 (binding mainly in cortical and cerebellar regions).

We have proposed a different interpretation, largely based on pharmacological facts and supported by experimental results. Thus it was shown several decades ago that 5-HT_1A_ receptors can exist in a high or a low-affinity state, depending on whether they are coupled or not to their G proteins^61,62^. This property implies that whereas 5-HT_1A_ receptor antagonists bind to the total pool of receptors, 5-HT_1A_ receptor agonists preferentially bind to a subpopulation of receptors in their high-affinity state^63^. Consequently, the radiolabelled antagonists, i.e. the current PET radiopharmaceuticals, allow PET neuroimaging of the total pool of receptors, regardless of their functional state. In contrast, and according to this pharmacological mechanism, agonists bind preferentially to the functionally active state of receptors (see Fig. 1).

### [^18^F]-F13640, a new radiopharmaceutical validated in human subjects

In order to specifically target 5-HT_1A_ receptors in their G-protein-coupled functionally active state, we have developed a highly selective 5-HT_1A_ receptor agonist, [^18^F]-F13640^64^. F13640, also known as befiradol or NLX-112, is a potent ligand with nanomolar affinity at 5-HT_1A_ receptors, and exceptional selectivity over a wide range of more than fifty receptor and transporter subtypes^65,66^. Its pharmacology has been extensively investigated, showing that it efficaciously activates 5-HT_1A_ receptors both in vitro and in vivo. Its specificity for 5-HT_1A_ receptors in vivo is supported by the observation that its effects in various experimental models are abolished by co-administration of the 5-HT_1A_ receptor antagonist, WAY100635.

The use of [^18^F]-labelled F13640 as an agonist PET tracer for functionally active 5-HT_1A_ receptors was recently investigated and validated in animal models (rat, cat and non-human primate). The results showed that the in vivo distribution pattern of [^18^F]-F13640 is very different from that of an antagonist radiotracer, e.g. [^18^F]-MPPF. There were also striking distribution differences for [^18^F]-F13640 between in vitro and in vivo results in rat brain. A probable explanation for the distinct binding patterns between 5-HT_1A_ agonist and antagonist radiotracers could be that the ratio of G-protein-coupled versus uncoupled receptors might be much lower in vivo compared to in vitro findings. In several animal species, although the blockade of labelling by the antagonist WAY-100635 was incomplete, [^18^F]-F13640 binding was almost completely blocked by the agonist 8-OH-DPAT. This may be due to a more effective competition between agonists as they both compete for receptors in the high-affinity state only. Notably, in vitro [^18^F]-F13640 binding was markedly reduced by Gpp(NH)p, an agent that switches receptors into an uncoupled state, demonstrating that [^18^F]-F13640 binds preferentially to G-protein-coupled 5-HT_1A_ receptors, a property that is consistent with its agonist activity^64^. In contrast, uncoupling of 5-HT_1A_ receptors from G proteins tends to increase the binding of the antagonist [^18^F]-MPPF, as previously reported^67,68^. These results highlight the fact that although both [^18^F]-F13640 and [^18^F]-MPPF are selective 5-HT_1A_ receptor radioligands, they bind to different pools of receptors depending on their coupling state. This proof of concept of the specific binding of [^18^F]-F13640 to functionally active receptors was supported by a post-mortem study in Alzheimer subjects^68^. This study showed that the in vitro binding of [^18^F]-F13640 differs from that of [^18^F]-MPPF, the prototypical radiopharmaceutical. [^18^F]-F13640 is therefore a promising PET tracer for in vivo imaging and quantification of functionally active 5-HT_1A_ receptors in the human brain, and a first-in-man study with this radiopharmaceutical is currently underway (study registration: EudraCT 2017-002722-21). The first human in vivo brain images with [^18^F]-F13640 showed a binding pattern different from that seen with conventional antagonist 5-HT_1A_ radiopharmaceuticals^69^. Although the binding regions were well correlated with the density of 5-HT_1A_ receptors, the regions of interest were mainly cortical, indicating that these brain regions are enriched in functionally active receptors. The [^18^F]-F13640 binding in cerebellum is less frequent, since this region is known to be poor in 5-HT_1A_ receptors, although PET studies in volunteers have revealed the presence of 5-HT_1A_ receptors in the cerebellar vermis of the cerebellum^70^.

## PET imaging of functionally active 5-HT_1A_ receptors: how to revisit this receptor in schizophrenia?

The development of [^18^F]-F13640 as an agonist PET radiopharmaceutical opens new opportunities for nuclear medicine. Until now, in vivo exploration of 5-HT_1A_ receptor in schizophrenia has mostly focused on the total pool of receptors with the default use of antagonist PET radiopharmaceuticals. We propose that the specific exploration of “functional” 5-HT_1A_ receptors, coupled to their G proteins, opens the way to revisiting the in vivo exploration of this receptor. Comparison between agonist and antagonist PET radiotracer binding could provide a promising and unprecedented receptor mapping strategy, potentially revealing new pathophysiological mechanisms. The availability of a radiopharmaceutical able to measure 5-HT_1A_ agonist sites, enables the undertaking of different protocols to explore 5-HT_1A_ receptors in schizophrenia: either using [^18^F]-F13640 alone, or by comparing the labelling of [^18^F]-F13640 with that of [^18^F]-MPPF or [^11^C]-WAY1006365. By opening the possibility to specifically explore functionally active receptors instead of total receptor densities, use of [^18^F]-F13640 could help to investigate issues that have remained unresolved by previous neuroimaging protocols.

### Revisiting pathophysiological processes

Protocols based on the concept of functionally active receptors may help to better define how variations in the expression of 5-HT_1A_ receptors could represent a biological trait marker of schizophrenia. First, comparing subjects with schizophrenia with healthy controls matched for sex and age could bring greater precision in the mapping of receptor densities. This could help to clarify the contradictory results previously found in the limbic and frontal cortices in post-mortem and in vivo studies, notably because the pronounced cortical distribution of [^18^F]-F13640 binding is favourable to the exploration of these brain areas. As a next step, the identification of new functionally active receptor patterns could be associated to cognitive status and illness state. The question of the presence of depression in schizophrenia, considered by some authors as more than a comorbidity^71^ could also be explored, leading to more subtle phenotyping of different populations of schizophrenic patients. In particular, these patterns could also be linked to suicidal behaviour for which 5-HT_1A_ receptor status is a key player^72^, life-time prevalence of suicide being around 5% in patients with schizophrenia^73^. Other notable clinical presentations such as catatonia that, according to the DSM-5, is not linked as a subtype to schizophrenia anymore, could be explored through the prism of the functionally active receptor pattern. As gender differences have been little studied so far or only provided preliminary results that deserved further investigation^39,40,42^, additional analysis could also be carried out concerning this question. Such protocols should be extended to schizophrenia patients’ relatives, since this disorder is defined by a high heritability and genetic vulnerability^28^. Finally, genetic polymorphisms of the 5-HT_1A_ receptor, as described earlier, could be at the origin of variation in the density of functionally active receptors and in treatment response to antipsychotics. All these neuroimaging protocols and the extension of this functionally active receptor approach to other neurotransmitters, will undoubtedly strengthen the concept of Research Domain Criteria (RDoC) proposed as an alternative method of classification in psychiatry with more emphasis on neurobiology^74^.

### Revisiting pharmacological mechanisms

The use of protocols based on the concept of functionally active receptor would also provide new insights into the understanding of pharmacological responses. In a previous PET study performed with the radiolabelled antagonist [^11^C]WAY100635, no difference in total 5-HT_1A_ receptor densities was found in schizophrenia patients before and after treatment with the antipsychotic ziprasidone, but there was a significant association between 5-HT_1A_ binding and improvement in negative symptoms^51^. Longitudinal studies based on the examination of functionally active receptor densities before and after treatment with antipsychotic drugs could revisit these previous results and show some changes in receptor patterns. Do drug-resistant subjects have a specific pattern of functionally active receptors? Do these receptors change differently over the course of treatment, depending on its efficacy? These protocols will be demanding because of the possible 5-HT_1A_ receptor occupancy rate of some antipsychotics having affinity for this receptor. To avoid such potential confounding factors related to drug occupancy, it may therefore be advisable to initially focus investigation on the effects of antipsychotics having a weak affinity for 5-HT_1A_ receptors. These results could then be correlated to the treatment response rate and may define new profiles between the responders and the non-responders. Moreover, there may be distinct patterns of functionally active receptor densities in drug-resistant patients.

### Integrating new multimodal imaging approaches

The literature in biological psychiatry is rich in clinical and research studies that have been performed using different imaging modalities on both separate positron emission tomography (PET) and magnetic resonance (MR) scanners. However, the current emergence of PET/MR cameras could broaden this approach^75^. While neurologists already make extensive use of this hybrid imaging modality, there is little research currently published in psychiatry using PET/MR. Although PET is able to quantify receptor densities, receptor occupancy by antipsychotics, and, with the development of agonist PET tracers such as [^18^F]-F13640, also quantify receptors specifically coupled to their G proteins, this approach cannot measure dynamically functional information. On the contrary, functional MRI offers the opportunity to map changes in cerebral activity occurring at resting state or during cognitive tasks and after administration of drugs; but it lacks mechanistic specificity as it provides no information on the receptors primarily targeted^76,77^. Combination of regional patterns of 5-HT_1A_ functionally active receptors, functional connectivity, and grey matter volume, derived from the disease characteristic networks, can potentially discriminate individual patients with different phenotypes. By analysing the functional connectivity of resting-state fMRI data in schizophrenia, the cortico–cerebellar–striatal–thalamic loop abnormalities, classically described could be correlated with the cortical binding of [^18^F]-F13640, which in turn could be associated with the cognitive performance of the subjects. Here again, the possibility of longitudinal follow-up, and thus intra-individual comparisons, would allow a distinction to be made between those subjects who respond and those who do not respond to pharmacological or psychotherapeutic strategies.

## Conclusions

The exploration of 5-HT_1A_ receptors in psychiatry has given rise to numerous studies. Although this receptor is associated with the pathophysiological mechanisms of schizophrenia and the pharmacological mechanisms of new-generation antipsychotics, its characterization remains incomplete. The contributions of molecular imaging, first in vitro on brain tissue, then in vivo, thanks to PET imaging, have not permitted significant advances but the recent concept of PET neuroimaging of G-protein-coupled receptors makes it possible to revisit PET brain exploration by enabling totally new research paradigms. These will make it possible to follow changes in the pattern of functionally active 5-HT_1A_ receptors in relation to disease symptomatology, such as cognitive dysfunction or mood deficits, both to better distinguish phenotypes and also to understand the basis of the therapeutic response to antipsychotic drugs. It is likely that the strategy of targeting functionally active receptors, as described above for the 5-HT_1A_ receptor, could also be usefully extended to other crucial receptors in pathophysiology or therapy of schizophrenia.
